# Anthrometric dimensions and their impact on cardiovascular risk factors

**DOI:** 10.1097/MD.0000000000038140

**Published:** 2024-05-24

**Authors:** Zekiye Gözde Kara, Doğuş Özdemir Kara

**Affiliations:** a Department of Autopsy, Turkish Council of Forensic Medicine Ankara Head Office, Ankara, Turkey; b Department of Pathology, Turkish Council of Forensic Medicine Ankara Head Office, Ankara, Turkey.

**Keywords:** abdominal adipose tissue, atherosclerosis, autopsy, cardiac risk factors

## Abstract

Central obesity is an important risk factor for cardiovascular disease. The abdominal subcutaneous adipose tissue thickness (ASATT) can be used to evaluate central obesity. The objective of this study was to compare ASATT with cardiovascular risk factors and other anthropometric parameters to show that ASATT can be a useful tool for the early assessment of heart disease risk. In this observational cross-sectional study, anthropometric measurements of 100 autopsied decedents, including waist circumference, hip circumference, waist/height and waist/hip ratio, aortic outlet and coronary artery atheroma plaque densities, heart weight, ventricular wall thickness, and ASATT, were assessed. The research data were evaluated using the Statistical Package for the Social Sciences for Windows 25.0. The average ASATT of the male group was 40.36 mm (SD: 11.00), and the average of female cases was 46.34 mm (SD: 18.12). There was no statistically significant difference between the sexes and both age groups in terms of the ASATT score (*P* > .05). There was a positive correlation between ASATT and waist circumference, hip circumference, and waist/height ratio in both sexes (*P* < .05). While ASATT was not related to atheroma density in the coronary arteries of men (*P* > .05), it was correlated with atheroma density in all 3 coronary arteries of women (*P* < .05). In the male group, the aortic inner surface atheroma density was positively correlated with ASATT (*P* < .05). In both sexes, there was a positive correlation (*P* < .05) between ASATT and heart weight; however, no such correlation was observed with right and left ventricular wall thickness (*P* > .05). ASATT is related to other anthropometric measurements, atherosclerosis of critical vessels, and heart weight, and can be used to scan the patient population for heart disease risk assessment with noninvasive methods.

## 1. Introduction

Worldwide, cardiovascular diseases (CVD) are the most common cause of mortality and morbidity, with 80% of cases occurring in developing countries. Studies conducted until the middle of the twentieth century identified smoking, hypertension, diabetes, and dyslipidemia as independent risk factors for CVD.^[1]^

Coronary atherosclerosis is the most common cause of death in patients with cardiovascular diseases. Atherosclerosis is a chronic vascular inflammation resulting from interactions between cardiovascular risk factors and arterial vessel walls.^[2]^ In particular, dyslipidemia and inflammation are 2 interrelated determinants in the process of atherosclerosis.^[3]^ Owing to the interaction between atherosclerosis and dyslipidemia, one of the most easily measured and controlled atherosclerosis risk factors is obesity, which is characterized by excess fat accumulation in the body.

Body mass index (BMI) is used to determine the level of obesity. Some studies have shown that, in addition to excess adipose tissue, its distribution in the body affects the risk of CVD.^[4]^ Studies have shown that central obesity is a better predictor than BMI for CVD risk assessment. Individuals with a normal BMI and a broader waist circumference compatible with central obesity have a higher risk of cardiovascular disease than individuals with a normal waist circumference and the same BMI measurements.^[5]^

Abdominal subcutaneous adipose tissue thickness (ASATT) can be used to evaluate central obesity. Studies have shown that ASATT thickness allows accurate prediction of whole-body fat storage and regional adiposity in both men and women.^[6]^ A significant correlation was found between total adipose tissue mass and ASATT.^[7]^ Therefore, measures of regional subcutaneous adiposity, such as subcutaneous tissue thickness, are widely used in population studies to estimate body composition and, in particular, body fat mass.^[8]^ There are also studies suggesting an association between ASATT and cardiovascular risk factors.^[9]^

The objective of this study was to compare ASATT with arterial atherosclerosis density, heart weight, ventricular wall thickness, and other anthropometric parameters considered risk factors for cardiovascular disease, taking into account the change in sex and age, and to point out that ASATT can be used to detect heart disease risk factors at an early stage.

## 2. Material and method

In this observational cross-sectional study, anthropometric measurements of 100 autopsied decedents, including waist circumference, hip circumference, waist/height and waist/hip ratio, aortic outlet and coronary artery atheroma plaque densities, heart weight, ventricular wall thickness, and ASATT, were assessed.

Between July 1, 2019 and January 31, 2020, a total of 1484 autopsies were performed in our institution, of which 348 were female and 1136 were male. Among these cases, 100 decedents aged between 25 and 65 years were included in the study.

The decision to perform an autopsy in all these cases was taken by the prosecutor’s office, and their deaths were suspicious, unexpected (cases found dead at home), sudden and/or initially unexplained (cases without a previous diagnosis of the disease), or problematic (accidents, suicides, homocide).

Cases that started to decay; cases with traumatic findings in the organ vessels that were measured in our study; morbidly obese or cachectic cases; and cases with congenital height, weight, or organ anomalies were not included in the study.

The corpses are kept in refrigerated cabinets before the autopsy begins. Some of the measurements (height, waist circumference, hip circumference) were made before the autopsy, some were made (ASATT) just after the autopsy started, and some were made (heart weight, ventricle wall thickness, aorta atheroma plaque density) during the autopsy. All these measurements took about half an hour in the autopsy room. However, these procedures were performed without interrupting the autopsy routine. Informed consent was obtained from the relatives of the deceased for the anthropometric measurements. The autopsied cases were handed over to their relatives after the autopsy was completed.

The anthropometric measurements were 1-waist circumference, 2-hip circumference, 3-waist/height ratio, and 4-waist/hip ratio, and cardiovascular disease risk factors were 1-coronary artery atheroma plaque densities, 2-aortic outlet atheroma plaque densities, 3-heart weight, and 4-wall thickness of both ventricles.

Two age groups (25–44 years: Group 1, 45–65 years: Group 2) were formed in male and female cases, 25 to 44 and 45 to 65.

The relationship between the decedents’ ASATT, the anthropometric measurements given above, and cardiovascular risk factors was evaluated by adding age and sex parameters. In the study, the relationship between the variables and different age groups and different gender groups was analyzed.

ASATT was measured in millimeters (mm) from the thickest part of the subcutaneous area, 1 cm below the umbilicus, with an Astor digital caliper after the skin was removed during autopsy. The measured range was defined as the area between the skin and lamina profunda of the fascia superficialis (subcutaneous fat tissue area).

Other anthropometric measurements were made in centimeters (cm) using a tape rule with the cases in the supine position. Height was measured as the vertex-heel distance, waist circumference was measured from the parallel line passing through the umbilicus, and hip circumference was measured as the greatest width of the femur at the level of the greater trochanters.

Heart weight was measured and recorded with a digital scale used in the autopsy room. After the heart was appropriately opened, right and left ventricular wall thicknesses were measured using a tape rule, 1 cm below the tricuspid valve on the right and 1 cm below the mitral valve on the left, excluding the trabecular structure.

The ratios of coronary vessel atheroma plaques in the cases were evaluated via examination of hematoxylin and eosin-stained slides by a pathologist under a light microscope. We created 6 groups: no atheroma: normal, 0% to 10%: minimal, 10% to 25%: mild, 25% to 50%: intermediate, 50% to 75%: advanced, and >75%: intensive.

The aorta was examined after the lumen was opened longitudinally. According to the percentage of atheroma plaques macroscopically covering the lumen, we created 6 groups: no atheroma: normal, 0% to 10%: minimal, 10% to 25%: mild, 25% to 50%: intermediate, 50% to 75%: advanced, and >75%: intensive.

The research data were evaluated using the Statistical Package for the Social Sciences for Windows. Descriptive statistics were presented as mean (±) standard deviation, median (minimum–maximum), frequency distribution, and percentage. The Kolmogorov–Smirnov test was used to analyze whether the data were normally distributed. Since the data were not normally distributed, the Mann–Whitney U test was used for pairwise comparisons, the Kruskal–Wallis test was used for comparing more than 2 variables, and the Spearman correlation test was used to determine the relationship between variables. The results were evaluated at a 95% confidence interval, and the significance level was set at *P* < .05.

The Spearman test was used to look at the correlation of intrasexual variables (waist circumference, hip circumference, waist/height and waist/hip ratio, aortic outlet and coronary artery atheroma plaque densities, heart weight, ventricular wall thickness) with subcutaneous adipose tissue thickness (as shown in Tables 4 and 5). The reason for examining intrasexual correlations separately in this way is the difference between the number of male and female cases, which is one of the limitations of our study. Spearman test was used to look at the relationship between the variables, but Kruskal–Wallis was applied as a multivariation test to determine whether there was a significant difference.

For the conduct of this study, permission was issued by the review board and ethics committee of the Turkish Council of Forensic Medicine dated May 20, 2019 and numbered 21589509/2019/412.

The private and personal data of decedents that support the findings of this study are available from our institution, but restrictions apply to the availability of these data, which were used under the license for the current study and so are not publicly available. However, the data are available from the authors upon reasonable request and with permission from our institution. In addition, the overall analysis and main variables can be requested from the author for review. Care was taken to protect the personal data of the patients, and informed consent was obtained from the relatives of the deceased for the anthropometric measurements for the study.

## 3. Results

Between July 1, 2019 and January 31, 2020, a total of 1484 autopsies were performed in our institution, of which 348 were female and 1136 were male. Among these cases, 100 decedents aged between 25 and 65 years were included in the study. Of the 100 decedents included in the study, 75 (75%) were male and 25 (25%) were female.

The mean decedent age was 48.37 (SD: 12.49). The mean age was 43.92 for women and 49.85 for men. Twelve (48%) of the women included in the study were aged 25 to 44 (Group 1) years, while thirteen (52%) were aged 45 to 65 (Group 2) years. Among men, 23 (30%) were in the age range of 25 to 44 years, while 52 (70%) were in the age range of 45 to 65 years. The age distribution of the cases according to gender is shown in Table 1.

**Table 1:** Age and sex distribution of the cases.

Gender	Age			
	25–44 ages		45–65 ages	
	n	Percentage	n	Percentage
Male	23	65.7	52	80.0
Female	12	34.3	13	20.0
Total	35	100	65	100

Among the female decedents, 2 were nullupars, and 23 had a history of at least 1 pregnancy.

The mean ASATT of the examined cases was 41.86 mm (SD: 13.30), while the mean ASATT of male cases was 40.36 mm (SD: 11.00), and the mean ASATT of female cases was 46.34 mm (SD: 18.12). There was no statistically significant difference between the sexes and both age groups according to the ASATT (*P* > .05).

The mean waist circumference (WAC) of the cases was 94.87 cm (SD: 13.53). The mean WAC for men was 96.21 cm (SD: 13.23), and the mean WAC for women was 90.84 cm (SD: 13.87), respectively. There was no statistically significant difference between the sexes in terms of the WAC. However, WAC increased with age.

The mean hip circumference (HC) of all cases was 102.21cm (SS: 10.01). The mean HC of the men group was 101.75cm (SS: 9.85), and the mean HC of the women group was 103.58cm (SS: 10.54). There was no statistically significant difference between the sexes in terms of HC (*P* > .05).

When the interrelationships of ASATT, waist/height and waist/hip ratio were analyzed, it was observed that ASATT increased in relation to waist/height and waist/hip ratio. Increasing waist/height ratio also increased waist/hip ratio (*P* < .05). (Table 2)

**Table 2:** The correlation of abdominal subcutaneous adipose tissue thickness with the waist/height and waist/hip ratios for both genders. Kruskal–Wallis was applied as a multivariation test to determine whether there was a significant difference.

		Subcutaneous fat	Waist/height ratio	Waist/hip ratio
Subcutaneous fat (mm)	Correlation coefficient	1.000	.673*	.405*
	Sig. (2-tailed)	.	.000	.000
	N	100	100	100
Waist/height ratio	Correlation coefficient	.673*	1.000	.697*
	Sig. (2-tailed)	.000	.	.000
	N	100	100	100
Waist/Hip Ratio	Correlation coefficient	.405*	.697*	1.000
	Sig. (2-tailed)	.000	.000	.
	N	100	100	100

* Correlation is significant at the 0.01 level (2-tailed).

The mean height of the cases was 168.86 cm (SD:8.46), the average of men was 171.59 cm (SD:6.96), and the average of women was 160.68 cm (SD:7.27).

The mean waist/height ratio (WAHER) of the total cases was 0.562 (SD: 0.082), the mean WAHER was 0.561 (SD: 0.078) in men, and the mean WAHER was 0.567 (SD: 0.094) in women. While there was no statistically significant difference between sexes in the waist/height ratio (*P* > .05), WAHER rise was detected with increasing age among different age groups, and this was statistically significant (*P* < .05).

Examining the waist/hip ratio (WAHIR) of the cases, the mean WAHIR was 0.92 (SD: 0.07) and the mean WAHIR was 0.94 (SD: 0.06) in the men group and 0.87 (SD: 0.08) in the women group. WAHIR increased with age, and there was a statistically significant difference between the sexes (*P* < .05).

As the atheroma density of the inner surface of the aorta was evaluated, there was a statistically significant difference between the sexes, and the atheroma density was higher in the male group (*P* < .05). In addition, among the age groups, atheroma density accelerated in older age group 2 (*P* < .05).

The heart weights of the cases were between 180 and 715 g, and the average heart weight was 419.10 g (SD: 107.19). The heart weight rises with increased age. Although there was no statistical difference between the right ventricular wall thickness and age groups, it was found that the left ventricular wall thickness increased with increasing age, and this difference was statistically significant (*P* < 005).

When we analyzed the relationship between ASATT and anthropometric parameters, there was a positive correlation (*P* < .05) with WAHER, WAHIR, WAC, HC, and heart weight in men (Table 3).

**Table 3:** The relationship of anthropometric measurements and cardiac parameters with abdominal subcutaneous adipose tissue thickness of men and women. “+” means correlation exists with variable, “−” means that there is no correlation with variable comparing with different genders.

	Men	Women
Waist/height percentage	+	+
Waist/hip percentage	+	−
Heart weight	+	+
Right/left ventricular wall thickness	−	−
Waist circumference	+	+
Hip circumference	+	+

Considering the relationship between ASATT and anthropometric parameters in women, there was a positive correlation with WAHER, WAC, and HC, similar to that in male decedents (*P* < .05). However, no such relationship was found for WAHIR (*P* > .05) (Table 3).

In terms of ASATT impact on coronary artery atherosclerosis, there was no correlation between the left descending coronary artery and the circumflex artery (*P* > .05), while there was a moderate correlation with the right coronary artery (*P* < .05). As this relationship was interpreted between the sexes, ASATT was not associated with atheroma density of the coronary arteries in the male group (*P* > .05) (Table 4), whereas atheroma plaque density of the coronary arteries increased as ASATT advanced in the female group (*P* < .05). This aspect was different for the aortic inner surface atheroma density. There was a positive correlation (*P* < .05) with ASATT in the male group, and this relationship was most intense in the moderate atheroma plaque density subgroup; however, no significant correlation was found in the female group.

**Table 4:** The correlation of abdominal subcutaneous adipose tissue thickness with the coronary arteries of men. The Spearman test was used to look at the correlation of intrasexual variables with subcutaneous adipose tissue thickness.

			LAD	RCA	Cx	Subcutaneous fat thickness (mm)
Spearman rho	LAD	Correlation coefficient	1000	.542*	.547*	.216
		Sig. (2-tailed)		.000	.000	.062
		N	75	75	75	75
	RCA	Correlation coefficient	.542*	1000	.653*	.205
		Sig. (2-tailed)	.000		.000	.078
		N	75	75	75	75
	Cx	Correlation coefficient	.547*	.653*	1000	.177
		Sig. (2-tailed)	.000	.000		.128
		N	75	75	75	75

RCA = the right coronary artery.

* Correlation is significant at the 0.01 level (2-tailed).

In both sexes, the heart weight increased with the increase in ASATT; however, there was no correlation between right and left ventricular wall thickness and ASATT (*P* > .05) (Table 5).

**Table 5:** The correlation of abdominal subcutaneous adipose tissue thickness with the heart weight, right and left ventricular wall thickness in women. The Spearman test was used to look at the correlation of intrasexual with subcutaneous adipose tissue thickness. The reason for examining intrasexual correlations separately in this way is the difference between the number of male and female cases.

			Subcutaneous fat thickness (mm)
Spearman rho	Heart weight (gr)	Correlation coefficient	.459^*^
		Sig. (2-tailed)	.021
		N	25
	Right ventricular wall thickness (cm)	Correlation coefficient	−.111
		Sig. (2-tailed)	.598
		N	25
	Left ventricular wall thickness	Correlation coefficient	−.008
		Sig. (2-tailed)	.968
		N	25

When the final reports of the causes of death of the cases were analyzed, 31 (31%) of the cases died from atherosclerotic heart disease, 17 (17%) from general body trauma, 13 (13%) from firearm injuries, 9 (9%) from hanging, 6 (6%) from intoxication (carbon monoxide, methyl alcohol, etc), and 9 (9%) from other causes (tamponade, aortic dissection, breast cancer, etc). In 15 (15%) cases, the cause of death could not be determined and was referred to the Forensic Medicine Institution (the relevant Istanbul specialized board). In other words, the cases in the group formed had different backgrounds and causes of death, reflecting the social structure.

## 4. Discussion

In 2005, a study conducted to determine the anthropometric measurements of the Turkish population revealed that the average height and weight of men were 168.88 cm and 74.74 kg, respectively. The mean of the same measurements in women was found to be 155.03 cm and 67.12 kg, approximately.^[10]^ When this study was compared with previous anthropometric studies conducted in Turkey, it was found that both weight and height increased in both sexes over the years. It is noteworthy that this increase continued in our study, and the average height of males reached 171.59 cm and the average height of females reached 160.68 cm. In addition, in our study, WAC was found to increase in both sexes, and HC was slightly increased compared to the aforementioned study. This can be attributed to socioeconomic, nutritional, and health conditions that have developed over the years.

In a study of 1100 female cases from south-eastern Turkey, 57.6% of the cases were overweight and 24.1% of the cases were obese. In addition, a strong association was found between an increased number of pregnancies and being overweight.^[11]^ In our study, among the female decedents, 2 were nullupars and 23 had a history of at least 1 pregnancy.

In another study from Turkey, BMI values were higher in men than in women between the ages of 20 and 35 years, and BMI values were higher in women than in men afterwards. Especially after the 40s, the BMI of women was above 30.^[10]^ The reason why women are more prone to obesity than men in Turkish society (gender inequality in obesity) was found to be low education level, unemployment, and low income.

The incidence of obesity, which has been increasing worldwide since the 1980s, is at epidemic levels. Obesity is an independent cardiovascular risk factor in addition to enhancing cardiovascular risk factors such as dyslipidemia, type 2 diabetes, hypertension, and sleep disorders.^[12]^ Studies indicated that Turkey has the highest prevalence of obesity and type 2 diabetes in Europe.^[13,14]^

Dietary habits, a lack of physical activity, and other socioeconomic factors are reported to cause obesity. Particularly in Turkey, it is found in national surveys that 44% of daily energy is sourced only through bread, while 58% is sourced from bread and other grain derivatives. It is also reported that consumption of fruit and vegetables is not sufficient, and there is a higher tendency towards fast food consumption in urban areas, especially among children and adolescents (Turkish Ministry of Health, 2007 and 2002).^[15]^

The body mass index (BMI) is the most widely used anthropometric tool for assessing the relative body weight and classifying obesity. Nonetheless, when BMI is used alone, a result called paradoxic obesity occurs. That is, patients with chronic diseases and a high BMI have an inverse relationship, such as better survival time and a lower risk of cardiovascular events, compared with nonobese patients.^[16,17]^ Therefore, BMI alone cannot be used as an anthropometric marker in cardiovascular risk assessment.

As body composition differs between men and women; women have proportionally more fat mass and men have more muscle mass.^[18]^ And because muscle mass weighs more than fat mass, BMI can be misleading when assessing men and women for obesity.

In addition, recent data indicate that abdominal obesity (central obesity), determined by waist circumference, is a cardiovascular risk factor independent of BMI.^[12]^ Lately, some studies have pointed out that the local accumulation of adipose tissue is more important than total fat mass.^[19]^ Nevertheless, there is a strict association between central obesity (subcutaneous, intraabdominal, and visceral adipose tissue) and BMI. In one study, it was observed that the prevalence of central obesity increased when the BMI was above 30 kg/m^2^.^[20]^

Recently, the association between central obesity and other anthropometric parameters with different CVD risk factors or specific entities has attracted attention. For instance, in cases of heart failure with preserved injection fraction (HFpEF), increased WAC and WAHIR and their involvement with increased BMI were noted among the anthropometric parameters.^[21]^ Similarly, in our case series, a positive correlation was detected between WAC and WAHIR with ASATT, which we used to evaluate central obesity. In addition, the heart weights of these decedents increased with an increase in the ASATT score in both sex groups.

A study from India also reported an augmentation of direct CVD risk with an increase in WAC and WAHIR.^[22]^ In another study, diabetes, dyslipidemia, and increased right and left heart filling pressure were observed in female patients with central obesity when compared with the general advance in body weight.^[20]^ Moreover, in women, unlike men, a connection was revealed between WAC and fasting glucose, low-density lipoprotein cholesterol, and injection HFpEF.^[20]^ In our study, while there was no statistically significant difference between sexes in terms of the WAC, the WAC increased with age. However, there was a positive correlation between ASATT and WAC in both sexes.

Regional fat accumulation is thought to play a role in the pathophysiology of HFpEF with decreased left ventricular compliance and cardiopulmonary fitness, increased local and systemic inflammation, and neurohormonal mechanisms.^[21]^ Obesity feeds the atherosclerosis process, and they are 2 entities that have something in common. In both entities, lipids, oxidized LDL, and free fatty acids initiate the disease by activating inflammation.^[23]^ In addition, adipocytokines secreted from adipose tissue accelerate every stage of atherosclerosis by causing insulin resistance, hyperquagability, endothelial dysfunction, and increased inflammation.^[24]^ Local fat deposits act as endocrine organs, conducting the heart, blood vessels, liver, and skeletal muscle.^[16]^

In our study, a scaling-up density of atheroma plaque in the coronary arteries was observed in the female group as ASATT, which is an indicator of central obesity, increased. This was different when the inner surface atheroma density of the aorta was evaluated. There was a positive correlation between ASATT and atheroma density of the aorta in the male group, and this relationship was most intense in the moderate atheroma plaque density subgroup. However, there was no significant relationship in the women’s group. Another publication on anthropometric measurements showed a strong association between increased WAC and atheroma density in the carotid artery.^[25]^

Local fat deposition in the abdominal region may occur in subcutaneous or visceral areas. Some studies regard fat accumulation in the visceral area, while others emphasize that fat accumulation in the subcutaneous area is more important in terms of CVD risk.^[26,27]^ This may be attributed to possible genetic and ethnic differences.

When the importance of fat deposition in visceral organs is understood, imaging studies such as magnetic resonance imaging and tomography have been used to detect the amount of fat tissue content in any part of the body.^[28]^ As local fat deposition first starts in the subcutaneous area and then continues in the form of visceral deposition, qualifying subcutaneous adiposity with the ASATT can be a practical tool for prospective risk assessment.^[26]^

Generally, men have a propensity to accumulate fat in the abdomen (apple-shaped body type), while women have a lower-extremity fat mass (pear-shaped body type).^[18]^ A study from Japan contradicts this general idea of gender body shape. In this study, the muscle mass and subcutaneous adipose tissue thickness of men and women were compared in young (20–29) and old (70–79) age groups. In this study, subcutaneous fat tissue thicknesses from different parts of the body of women were found to be higher than those of men in both age groups.^[29]^ Our study revealed similar findings. Although there was no statistically significant difference between the sexes and both age groups in terms of the ASATT score, the average ASATT of the male group was 40.36 mm versus female cases (46.34 mm). In 2 studies from Turkey comparing male and female subjects in terms of central obesity, the rate of central obesity was higher in women than in men^[30,31]^ which was consistent with our findings.

One of the limitations of this study is the dominance of male cases, similar to other autopsy series. During the time period of our study, 76.5% of the autopsies performed in our institution were male, and this was consistent with the gender distribution in autopsies performed previously in Turkey.^[32]^ In a study from Nigeria, there was a similar male predominance of autopsy cases.^[33]^ In order not to bias the findings of the male predominance in the study, the relationship of female subjects with the variables was analyzed within the group. In the study, the relationship between the variables and different age groups and different gender groups was also analyzed.

The study is a cross-sectional quantitative study and the findings were evaluated and interpreted by both a forensic medicine specialist and a forensic pathologist. Our study is a multidisciplinary study that includes both macroscopic and microscopic findings.

## 5. Conclusion

The characterization of subcutaneous adiposity by ASATT may be a practical tool for prospective risk assessment, as local fat accumulation starts first in the subcutaneous region and then continues as visceral accumulation. Since our study showed a strong correlation between ASATT and anthropometric and CVD risk factors, we think that it can be used as a first step for heart disease risk assessment in patient populations by measuring ASATT with a cheaper and noninvasive method such as ultrasound.

The authors declare that they have no conflicts of interest. This study did not receive any specific grants from funding agencies in the public, commercial, or not-for-profit sectors.

## Acknowledgments

Special thanks to Professor Birol Demirel from Gazi Medical School, who supported and guided us during the whole study. All statistics were evaluated by Nazli Gökçe Kara, who is a professional statistician. Nazli Gökçe Kara.

## Author contributions

**Conceptualization:** Doğuş Özdemir Kara, Zekiye Gözde Kara.

**Data curation:** Zekiye Gözde Kara.

**Formal analysis:** Zekiye Gözde Kara.

**Investigation:** Zekiye Gözde Kara.

**Methodology:** Zekiye Gözde Kara.

**Project administration:** Zekiye Gözde Kara.

**Software:** Zekiye Gözde Kara.

**Supervision:** Doğuş Özdemir Kara.

**Validation:** Doğuş Özdemir Kara.

**Visualization:** Doğuş Özdemir Kara.

**Writing – original draft:** Doğuş Özdemir Kara.

**Writing – review & editing:** Doğuş Özdemir Kara.
